# Assessing Ecological Infrastructure Investments—A Case Study of Water Rights Trading in Lu’an City, Anhui Province, China

**DOI:** 10.3390/ijerph19042443

**Published:** 2022-02-20

**Authors:** Qiuyan Wang, Qingjian Zhao

**Affiliations:** College of Economics and Management, Nanjing Forestry University, Nanjing 210037, China; qiuyanwang0105@126.com

**Keywords:** ecological infrastructure, ecosystem services, water rights trading, water-saving irrigation, market clearing

## Abstract

Investment in ecological infrastructure construction alters behaviors and quantities of the ecosystem service (ES) provided, and market-clearing can learn the value and scale of ES. We integrated ecological and economic methods to put forward the idea of realizing the aquatic ecological products’ value by investing in water-saving irrigation infrastructure. Firstly, the demand for aquatic ecological products was calculated by a Cobb–Douglas production function, and then the supply of aquatic ecological products was estimated by InVEST and water-saving potential models; Finally, the scale of ecological infrastructure investment and the aquatic ecological products’ value are illustrated by cost-benefit analysis and market equilibrium theory. Research indicates that, (1) industrial water efficiency is high, and the ecological infrastructure construction provides a considerable number of ecological products; (2) implementing water-saving irrigation project is at least 30% more profitable than maintaining the status quo; (3) the market-clearing results showed that the market equilibrium price is about 0.256 USD/m^3^, and the transaction volume is about 1.667 billion m^3^. The output value of industrial enterprises after buying water can reach about 1.37 times of the current stage, reflecting the aquatic ecological products’ value. Investment in water-saving irrigation infrastructure has huge economic, social and ecological benefits, and provides reference for decision-making.

## 1. Introduction

With the rapid development of China’s economy and society, the high intensity of human activities and the low carrying capacity of the natural ecosystem have made the contradiction between human and ecology increasingly prominent [1]. “Clear waters and lush mountains are invaluable assets “powerfully expounds the dialectical unity relationship between natural environmental protection and socio-economic development. Protecting the ecology is conducive to promoting sustainable economic development [2]. At the same time, increasing the supply of ecological products can significantly increase the environment increment, enhance social well-being, and effectively meet the people’s growing demand for a better life. The concept of ecological products corresponds to the general ecosystem services [3,4]. It is a general term for the material products and services provided by the ecosystem, including provisioning services, regulation services and cultural services [5,6,7]. The research on ecosystem services mainly focuses on the following aspects: ecosystem service trade-offs and coordination methods [8,9], the temporal and spatial changes of ecosystem services [10,11,12,13,14], the formation mechanism and change driving factors of ecosystem services [15,16,17,18], the relationship between ecosystem services and human well-being [19,20], etc. Remote sensing and GIS technologies have been fully used in these researches of ecosystem services, and combined with different models to carry out large-scale spatial measurement of ecosystem service value, which has revolutionized the evaluation of ecosystem service value. Based on the reference of previous scholars’ research, this research combined the InVEST model and GIS platform to achieve an accurate value assessment and mapping of ecological products. As a product, ecological products emphasize their own value and the investment required to produce them. The realization of ecological product value is to internalize the value of ecological products with external benefits. In recent years, the theoretical and practical exploration of “ecological product value realization” has become a hot spot. Scholars have explored and proposed implementation mechanisms in terms of property rights, markets, compensation, and industrial models [21,22,23,24,25]. Some areas have carried out practical pilot projects for value realization, such as Lishui, Nanping, Fuzhou, etc. [26,27]. There are two main ways to realize the value of ecological products in research and practice [28]. One is the value realization path led by the government, including transfer payments, government purchases, ecological taxes, and ecological compensation; the second is a market-led value realization path, including ecological industrialization management, ownership trading, and green finance.

As an important part of ecological products, aquatic ecological products are indispensable and irreplaceable resources for the development of human society. With the gradual expansion of industrial scale, water demand is also increasing. However, in reality, it is difficult to increase supply by developing new water sources. Therefore, there is growing interest in investing in natural capital in the form of ecological infrastructure [29], with the purpose of obtaining lasting output of “ecosystem services”. From an economic perspective, it is necessary to conduct cost–benefit assessments beforehand, and market clearing can help understand the value of ecological products. Feasible ecological investment depends on [30]: (1) the value and demand for the services that ecological infrastructure provides; (2) a greater level of services after the ecological infrastructure construction; (3) and the ability, right, and willingness for an entity to make changes to an ecosystem, that is, to supply ecological infrastructure. Many studies are only the evaluation of ecosystem services. For example, Wong CP et al. [31] used a new interdisciplinary ecological production function to evaluate four ecosystem services in the Beijing Yongding River Green Ecological Corridor. Provides measurement standards such as the marginal value linking ecosystem characteristics and services; Labarraque D et al. [32] linked the loss of ecosystem services to the direct and indirect impacts of infrastructure construction, which expanded the scope of infrastructure project evaluation; Yang, M et al. [33] evaluated the changes in the value of ecosystem services on the basis of distinguishing ecological red line areas. Similar studies have failed to take into account the society’s demand for services generated by the construction of ecological infrastructure. When it comes to demand for ecosystem services, scholars mostly analyze from the perspective of whether the supply and demand of ecosystem services are balanced [34,35,36]. In terms of demand for aquatic ecological products, there are many studies on the prediction of water resources demand for regional economic development [37,38,39,40]. To make water resources meet the needs of economic development, water-saving irrigation infrastructure is gradually being developed and constructed, because farmland irrigation has greater water-saving potential. The research on the comprehensive benefits of farmland water-saving irrigation mainly involves the evaluation of water-saving potential [41,42,43] and the exploration of water-saving technology models [44,45]. The influencing factors of water-saving irrigation willingness to adopt technology [46,47,48] have also been paid attention to by scholars, mainly including personal characteristics, family characteristics, and policies.

Overall, viable ecological infrastructure investment requires comprehensive consideration of many aspects, rather than a single discipline. At present, there are relatively mature researches in each aspect, but few researches integrate them, only as Adamowicz W et al. conduct an ex-ante benefit–cost assessment and forecast market-clearing prices and quantities for ecological infrastructure investment contracts in the Panama Canal Watershed [30]. Therefore, this research is committed to integrating various disciplines and exploring the path to realize the value of ecological products. To invest in the construction of ecological infrastructure based on meeting one’s own needs, prior cost-benefit assessment is crucial, and understanding the future market size will help make correct decisions. This research will first make it clear that the demander can use aquatic ecological products to obtain greater benefits and is willing to bear a certain cost; secondly, after clarifying the supply side’s water resources, the supply curve is established; finally, the price and quantity of market clearing will be predicted through the theory of supply and demand equilibrium; Lu’an City, Anhui Province, China was used to verify the feasibility of the ecological infrastructure investment proposed in this research.

## 2. Study Area and Materials

**Figure 1:**
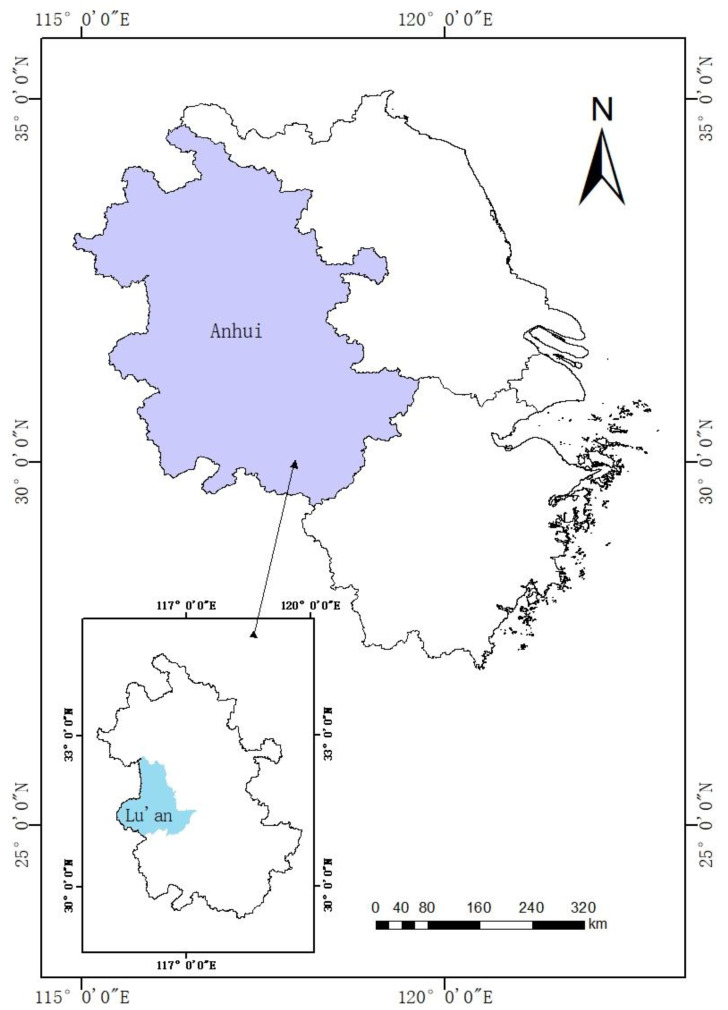
Map of Lu’an City.

Lu’an City is located in the west of Anhui Province and the west wing of the Yangtze River Delta. The city is between 115°20′ and 117°14′ east longitude and 31°01′ and 32°40′ north latitude (Figure 1), with a total area of about 15,451.2 square kilometers. Lu’an City has abundant surface water and numerous rivers. The total surface water resources are 8.366 billion m^3^. There are 6 main rivers in the territory, including Pihe, Shihe, Hangbuhe, etc. The Pishihang Comprehensive Utilization Project, built on the basis of the six major reservoirs of Foziling, Meishan, Mozitan, Xianghongdian, Longhekou, and Bailianya, is the largest artificial irrigation area in China and one of the seven largest artificial irrigation areas in the world. Its designed irrigation area is 730,000 hectares, of which Lu’an City has an irrigation area of 437,000 hectares. In early 2019, the Department of Water Resources of Anhui Province issued the “Anhui Province Water Right Confirmation and Registration Pilot Work Plan”. Jin’an District of Lu’an City became the first water right reform pilot area in the province and took the lead in carrying out the water right confirmation and registration work. Focusing on agricultural water rights, the rights of 173 culvert gates, 99 pumping stations, 268 reservoirs, and 344 ponds and dams have been confirmed to 257 rural collective organizations and major agricultural water users. A total of 900 water resources use rights certificates were issued, and the water volume was confirmed to be approximately 146.9 million m^3^, laying the foundation for water rights transactions. Therefore, this research selects Lu’an City, Anhui Province as the study area.

In this research, the socio-economic data mainly comes from the “Lu’an City Statistical Yearbook”, “Lu’an City Water Resources Bulletin”, “Anhui Province Farmland Water Conservancy Last Kilometer Special Plan (2018–2022)”, “Anhui Province Industrial Water Use Quota”, “Compilation of National Agricultural Product Costs and Benefits” and the official website of Anhui Provincial Department of Water Resources-Anhui Province Pishihang Irrigation District Administration Bureau, etc. The land use and precipitation data come from the Resource and Environmental Science and Data Center of the Chinese Academy of Sciences. The average annual potential evapotranspiration data comes from the National Earth System Science Data Sharing Service Platform. The soil data come from the Harmonized World Soil Database version 1.1 (HWSD) constructed by the Food and Agriculture Organization of the United Nations (FAO) and the Vienna International Institute of Applied Systems (IIASA). For other parameter values of the Water Yield module, refer to the InVEST model manual. Data such as water saving rate, yield increase rate, and construction cost of water-saving irrigation facilities are all derived from related published literature.

## 3. Methods

**Figure 2:**
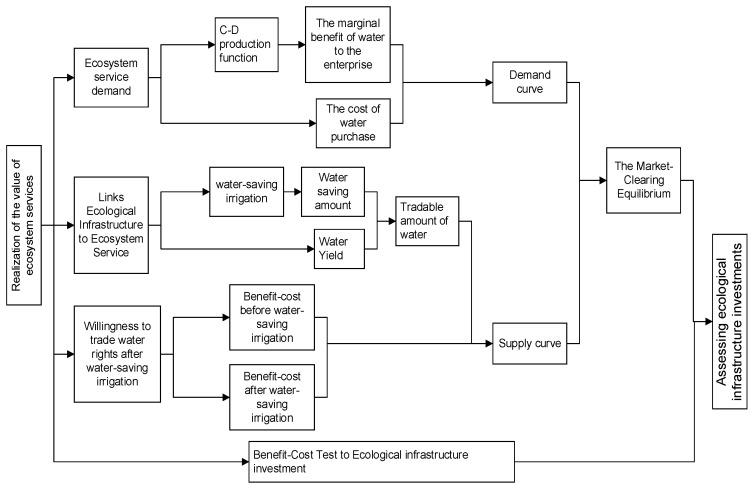
Research ideas.

We propose the general research idea that is shown in Figure 2: First, the demand for ecosystem services is clarified, that is, the enterprise’s demand for water, and it is assumed that the enterprise is willing to invest in the construction of ecological infrastructure. Secondly, the ecological infrastructure and services are linked, and the biophysical data that will be generated by the ecological infrastructure investment is clarified. Then, through investigation and cost-benefit analysis, the willingness of the entity to change the ecological infrastructure is obtained. Finally, the market is cleared based on the theory of equilibrium of supply and demand, the equilibrium price and quantity are understood, and the feasibility of the ecological infrastructure construction investment is clarified through the cost–benefit test.

The specific method is as follows:

### 3.1. C-D (Cobb–Douglas) Production Function

Understanding the marginal contribution of 1 m^3^ of water to the income of industrial enterprises will help provide the enterprise’s demand curve for water.

Industrial enterprises will create internal value by applying aquatic ecological products to production, processing, etc. By measuring the marginal benefits of industrial water use, the value of aquatic ecological products will be effectively internalized. Currently, most of the static methods (such as ROI and Payback Period, etc.) are used to measure the marginal benefits of industrial water use, ignoring the impact of technology and scale benefits, and there are certain limitations. In recent years, dynamic mathematical economic models have gradually been promoted and applied. The Cobb–Douglas (C-D) production function is derived by Cobb and Douglas, based on the impact of labor and capital input on output, the formula is as follows [49]:

(1) $$Y=AK^{\propto }L^{1-\propto }\;0\;\leq \;\propto \;\leq \;1$$

where Y represents industrial output value, A means technical level, K and L represent the amount of invested capital and the amount of labor, respectively, ∝ and 1−∝, respectively, represent the contribution degree of capital and labor to industrial output value.

Water is essential in the industrial production process, and the elastic price of water needs to be integrated into the calculation of the marginal benefit of industrial water. The calculation equation is as follows [50]:

(2) $$Y=A_{t}K^{\propto }L^{\beta }W^{\gamma }$$

where $A_{t}$ is a dynamic variable related to time, K and L represent the total investment in industrial fixed assets of the whole society and the number of industrial employees, respectively, W represents the total industrial water consumption, ∝,β, and γ represent the elasticity of capital, labor, and water, respectively.

Since the production function is non-linear, the logarithm of both sides of Equation (2) is taken, and the equation is:

(3) $$\ln Y=\ln A_{t}+\propto \ln K+\beta \ln L+\gamma \ln W$$

Taking the partial derivative with respect to W on both sides of Equation (3), the marginal benefit of industrial water ($R_{w}$) is obtained, and the result is the following equation:

(4) $$R_{w}=\frac{\partial Y}{\partial W}=\gamma \frac{Y}{W}$$

where $R_{w}$ represents the marginal benefit of water to industrial production, $\frac{Y}{W}$ represents the unilateral water output rate. Equation (4) shows that the marginal benefit of water is the reciprocal of water consumption per CNY 10,000 of output value multiplied by the elasticity of water.

In the production function, ∝+β+γ is regarded as the elasticity of scale. When ∝+β+γ>1, the return to scale increases, when ∝+β+γ=1, the return to scale remains unchanged, when ∝+β+γ<1, the return to scale decreases. In order to reasonably reflect the output contribution of water resources and eliminate the impact of scale elasticity, the conversion water elasticity coefficient is adopted. The calculation equation is as follows [50]:

(5) $$\gamma^{,}=\frac{\gamma }{\propto +\beta +\gamma }$$

Therefore, the marginal benefit equation of water is transformed into the following equation:

(6) $$R_{w}=\frac{\gamma }{\propto +\beta +\gamma }\cdot \frac{Y}{W}=\gamma^{,}\frac{Y}{W}$$

### 3.2. The Cost of Water Purchase

We propose the calculation equation for the cost of water purchase by enterprises is as follows:

(7) $$C_{q}=\frac{P_{q}Q_{q}+\varphi C_{g}}{Q_{q}}=P_{q}+\frac{\varphi C_{g}}{Q_{q}}$$

where $C_{q}$ represents the water purchase cost of the enterprise, $P_{q}$ represents the water purchase price of the enterprise (USD/m^3^), $Q_{q}$ represents the amount of water purchased by the enterprise (m^3^), $C_{g}$ represents the total cost of construction of water-saving irrigation facilities, φ represents the proportion of enterprise investment, which is included in the total government subsidies.

### 3.3. Water Yield

The water yield module of the InVEST model is based on the principle of water balance, and calculates water yield through parameters such as precipitation, plant transpiration, surface evaporation, root system depth, and soil depth. The calculation equation is as follows [51]:

(8) $$Y_{xj}=\left(1-\frac{AET_{xj}}{P_{x}}\right)\times P_{x}$$

where $Y_{xj}$ means annual water yield, $AET_{xj}$ represents the actual annual average evapotranspiration of a single unit x on land use type j, $P_{x}$ represents the average annual rainfall of a single unit x.

The module requires the following data: annual average rainfall, annual average potential evapotranspiration, soil depth, plant available water content, land use/land cover, watershed vector, biophysical table, and Z parameters.

### 3.4. Water-Saving Amount

On the premise of meeting the needs of agricultural development, the development of efficient water-saving irrigation methods for farmland can not only reduce the irrigation water quota for farmland in the planning year, but also increase the irrigation water utilization coefficient, thereby saving irrigation water [52]. The water-saving potential is mainly reflected in two aspects: the improvement of irrigation water utilization coefficient and the increase of efficient water-saving irrigation area [53]. The calculation equation of water-saving amount is as follows [54]:

(9) $$W_{jsaving}=\overset{3}{\underset{i=1}{\sum }}\left[A_{ij}\times q_{i}\times \left(\frac{1}{\mu_{0}}-\frac{1}{\mu_{it}}\right)\right]$$

where $W_{jsaving}$ represents the water-saving amount of farmland in the j_th administrative village, $A_{ij}$ represents the irrigated area of the j_th administrative village using the i_th irrigation technology, $q_{i}$ represents the irrigation water quota of crops under the i_th irrigation technology, $\mu_{0}$ represents the current year irrigation water utilization coefficient, $\mu_{it}$ represents the irrigation water utilization coefficient of the i_th irrigation technology in the planning year.

### 3.5. Tradable Amount of Water

Refer to the “Interim Measures for Market-oriented Transactions of Ecological Products in Dayang Town”: Generally, purchases are made at 2–8% of the value of the ecosystem product in the area where the project is located. Therefore, the tradable water volume in our research is defined as the sum of water-savings and 8% water yield. The calculation equation is as follows:

(10) $$W_{s}=W_{saving}+Y_{xj}*8\%$$

where $W_{s}$ represents the amount of tradable water, $W_{saving}$ represents the water-saving amount, and $Y_{xj}$ represents the annual water yield.

### 3.6. Farmland Irrigation Cost-Benefit Analysis before Water-Saving Irrigation

When water-saving irrigation is not used, farmland irrigation mainly relies on soil canal water delivery and flood irrigation. Leakage in the process of water delivery and loss of evaporation from flood irrigation have resulted in low irrigation water utilization [55]. A large amount of irrigation water is needed per cubic meter of farmland. The cost of farmland irrigation is mainly the water fee for irrigation, and the benefits are reflected in the output of crops. Therefore, the calculation equation of farmland irrigation cost-benefit is as follows:

(11) $$R_{nj}=P_{z}Q_{z}-P_{n}\times A_{j}$$

where $R_{nj}$ represents the farmland irrigation benefit of the j_th administrative village (dollars), $P_{z}$ represents the price of crops (USD/t), $Q_{z}$ represents the yield of crops per hectare (t/ha), $P_{n}$ represents the irrigation water fee for farmland (USD/ha), $A_{j}$ represents the crop planting area (ha) of the j_th administrative village.

### 3.7. Farmland Irrigation Cost-Benefit Analysis after Water-Saving Irrigation

The water-saving irrigation methods require high equipment investment costs and have the attributes of public goods. Therefore, in actual society, most of the government funds provide subsidies. This research adheres to the principle of “whoever benefits, who invests”, and the company is designed to be one of the main bodies of investment in water-saving irrigation equipment in the future. At this time, farmland irrigation costs mainly include water fees and construction costs of water-saving irrigation facilities; benefits include government and corporate subsidies, crop output values, and water rights transactions. The calculation equation is as follows:

(12) $$R_{nj}^{‘}=\overset{3}{\underset{i=1}{\sum }}Z_{i}A_{i}+P_{z}Q_{z}^{’}A_{j}+P_{mj}Q_{mj}-C_{g}-P_{n}^{’}A_{j}$$

where $R_{nj}^{‘}$ represents the farmland irrigation benefit (dollars) of the j_th administrative village after water-saving irrigation, $Z_{i}$ represents the government subsidy for the construction of the i_th water-saving irrigation facility (USD/ha). Include corporate investment in the total government subsidies. $A_{i}$ represents the irrigation area (ha) of the i_th water-saving irrigation facility, i=1, 2, 3 represent low-pressure pipeline water delivery, sprinkler irrigation, and drip irrigation, respectively. $P_{z}$ represents the price of crops (USD/t), $Q_{z}^{’}$ represents the yield of crops per hectare (t/ha) after water-saving irrigation, $P_{n}^{’}$ represents the farmland irrigation water fee after water-saving irrigation (USD/ha), $A_{j}$ represents the crop planting area (hectares) of the j_th administrative village. $P_{mj}$ represents the price at which the j_th administrative village is willing to sell water (USD/m^3^), $Q_{mj}$ represents the amount of water (m^3^) that the j_th administrative village is willing to sell.

### 3.8. Market Clearing Model

Market clearing refers to the state where the amount of willing demand and the amount of willing supply in the market are equal at a given price, which is an equilibrium state that eliminates excess supply or excess demand. The simple market clearing model assumes that the market is completely competitive, transaction costs are zero, and there are no uncertain factors affecting both supply and demand. The price mechanism is the only factor for market clearing and responds to the supply and demand situation. The equilibrium equation is:

(13) Dp=S(p)

where D represents the demand curve, S represents the supply curve, and p represents the price.

## 4. Results

### 4.1. Ecosystem Service Demand—Enterprise’s Demand for Water

The Cobb–Douglas (C-D) production function between the total industrial production value of Lu’an City and fixed asset input, labor, and industrial water is established according to Table 1. After taking the logarithm of both sides of the function, a linear regression analysis was established in SPSS (IBM, Armonk, NY, USA), and the confidence level of the regression coefficient of the T test was set to 95%.

The coefficients of determination R and $R^{2}$ obtained through the model are 0.971 and 0.944, respectively, indicating that the model has a high degree of goodness of fit. The F statistic is 22.279, and the significance test probability is 0.006, which is less than the significance level of 0.05, indicating that the equation passes the F test and is significant. The regression results are: the constant is 0.657; the regression coefficients of fixed assets, labor and industrial water are 0.97, 0.186 and 0.129, respectively. Therefore, the industrial water efficiency model of Lu’an City is as follows:

(14) $$Y=e^{0.657}K^{0.97}L^{0.186}W^{0.129}$$

Obviously, ∝+β+γ=1.285>1, indicating an increasing return to scale. The coefficient of elasticity of industrial water conversion is:

(15) $$\gamma^{,}=\frac{\gamma }{\propto +\beta +\gamma }=0.1004$$

According to Table 1, from 2010 to 2017, the average annual industrial water consumption was 320 million m^3^, and the average unilateral water output was 64.35 USD/m^3^. Then the marginal benefit ($R_{w}$) of industrial water use was 6.46 USD/m^3^, which means that an additional 1 m^3^ of water will increase the benefit of 6.46 dollars.

It is not practical for industrial enterprises to spend 6.46 dollars to purchase 1 m^3^ of water for production. The water-to-household price of industrial enterprises in Lu’an City (excluding sewage treatment fees) is 0.48 USD/m^3^. However, at such prices, industrial enterprises have insufficient water supply, so enterprises have the motivation to seek more water resources from outside, with the purpose of meeting production demand or scale expansion. Considering the huge potential for water saving in farmland irrigation, and the price of agricultural water is lower than the price of industrial water, industrial enterprises are willing to provide a feasible solution to farmland irrigation entities such as rural collective organizations: enterprises will bear the cost of water-saving irrigation facilities and require irrigation entities to implement farmland water-saving irrigation to save more water. At the same time, enterprises will purchase the saved water at a price higher than the price of agricultural water but lower than the price of industrial water. In China, most of the investment entities for the construction of water-saving irrigation facilities or farmland water conservancy projects are governments. If enterprises can invest in farmland water conservancy construction capital, they will simultaneously solve the water shortage problem of industrial enterprises and achieve multi-subject, multi-channel, and diversified investment channel goals.

In economics, as a “rational man”, an enterprise will pursue the maximization of profits, that is, the marginal cost is equal to the marginal benefit. Combining Equation (7), it can be seen that the relationship between the purchase price of water and the amount of water purchased is as follows:

(16) $$P_{q}=6.46-\frac{\varphi C_{g}}{Q_{q}}$$

From Equation (16), it can be seen that since part of the purchased water comes from water saved by water-saving irrigation, the amount and price of water that enterprises are willing to purchase are related to the construction cost of water-saving irrigation facilities. When the construction cost of water-saving irrigation facilities invested is higher, enterprises tend to trade at a lower price. Enterprises make decisions based on their own internal operating data.

### 4.2. Spatial Scope of Investment in Water-Saving Irrigation Infrastructure

**Figure 3:**
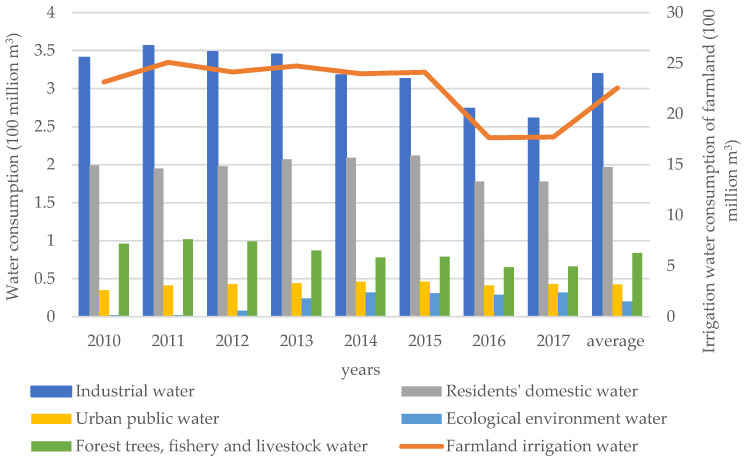
The utilization of water resources in Lu’an City from 2010 to 2017.

The construction of viable ecological infrastructure is to change the ecosystem by changing the biophysical quantity to provide services. Increasing the amount of water available for industry is a required ecological service. The use of water resources in Lu’an City from 2010 to 2017 is shown in Figure 3. Farmland irrigation is the largest user of water resources, followed by industrial water and domestic water. However, the economic benefits of agricultural water use are low and the consumption of water resources is large. The value of water resources can be improved if the agricultural water-saving water rights are transferred to industrial water. The “Opinions on Promoting the Comprehensive Reform of Agricultural Water Prices” promulgated by the State Council in 2016 pointed out: “Under the premise of meeting the requirements for agricultural water use in the region, we will implement cross-regional and cross-industry transfers of water-savings.” One of the driving forces for this transfer comes from the gap between industrial water prices and agricultural water prices. The price of agricultural water in Lu’an City does not exceed 0.015 USD/m^3^, while the price of industrial water to households (excluding sewage treatment fees) reaches 0.48 USD/m^3^. Judging from this price difference, the economic benefit per cubic meter of water from the transfer of agricultural water rights to industry is about USD 0.47. The economic benefits are very considerable.

The prerequisite for the transfer of agricultural water rights to industrial water is to ensure the basic water consumption for farmland irrigation. Only when there is a surplus in agricultural water consumption can the agricultural water rights be transferred to industry for a fee, so as to improve the value and efficiency of the use of water resources. The average water consumption of farmland irrigation in Lu’an City accounts for about 77.3% of the city’s average total water consumption. Under the premise that the proportion of water used in each part remains unchanged, if farmland irrigation saves 10% of water, the total water consumption can be saved by about 46%. Therefore, the implementation of farmland water-saving irrigation is conducive to the rational distribution and efficient use of water resources, and the paid transfer of water-saving water rights can economically encourage the farmers to take water-saving measures. In economics, as a “rational person”, farmers are pursuing the maximization of their own interests. The greater the benefits of water saving, the higher their enthusiasm for water saving. Technically, water-saving irrigation projects usually adopt measures such as channel lining, water pipes, sprinkler irrigation, and micro-irrigation to scientifically promote water-saving irrigation in farmland.

The entities of water-saving irrigation are mainly agricultural business entities such as village collective organizations, farmers’ professional cooperatives, professional households, family farms, etc., which use low-pressure pipelines for water delivery, sprinkler irrigation, micro-irrigation, and other high-efficiency water-saving facilities for water-saving irrigation. In addition to the costs borne by enterprises, the government will also provide certain water-saving irrigation subsidies to encourage water-saving irrigation. The specific subsidy standards are shown in Table 2.

**Figure 4:**
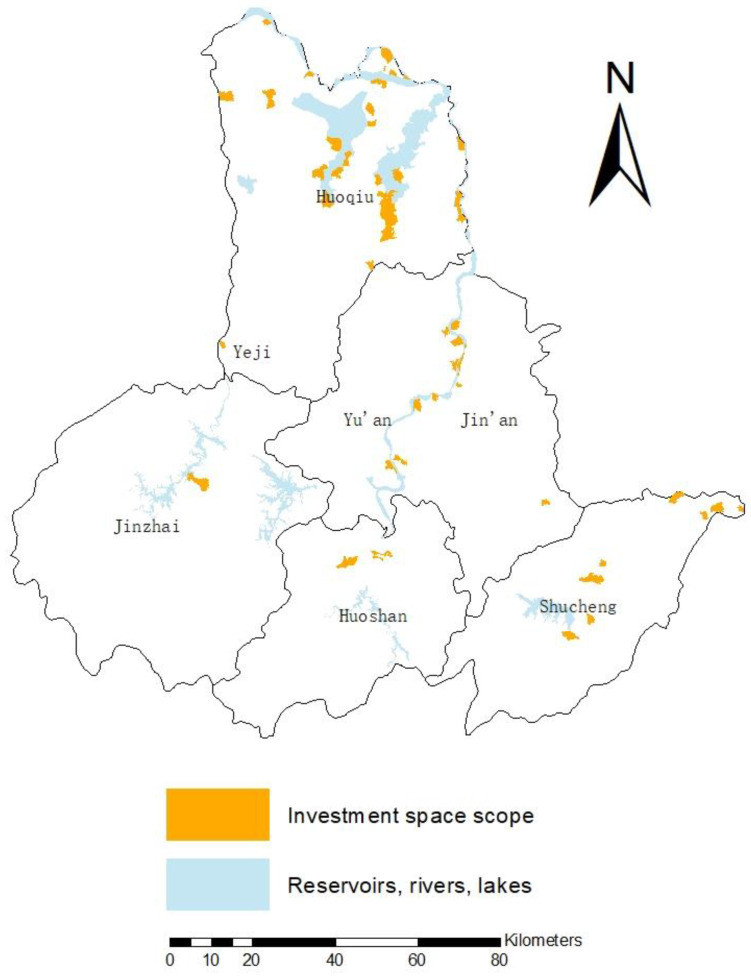
Distribution of cultivated land and water bodies in Lu’an City.

Combine the land use types and administrative village boundary maps in Lu’an City to make an area tabulation. The results show that there are 226 administrative villages whose water area is not zero. Except for reservoirs and lakes, water bodies account for up to 80.8% of the administrative village area. In total, 1701 administrative villages have arable land, of which 1232 administrative villages account for more than 50% of the arable land. Among the administrative villages with a water body area of more than 20%, there are about 52 administrative villages with a cultivated land area of not more than 50%. The distribution is shown in Figure 4. It can be seen that the administrative villages with cultivated land area less than 50% and water body area greater than 20% are mainly concentrated in Huoqiu County, and most of them are distributed along lakes, rivers or reservoirs. Other counties and districts also have sporadic distribution. Among them, there is only one in Yeji District, Jin’an District, and Jinzhai County, followed by Huoshan County with three, and Yu’an District and Shucheng County each with about 10 each. The 52 villages mentioned above can theoretically be the targets of water-saving irrigation investment.

Assume that all 52 administrative villages only use low-pressure pipeline water delivery, sprinkler irrigation, and micro-irrigation three water-saving facilities to irrigate 82% of the arable land in the administrative villages. (The “Last Kilometer” Special Plan for Farmland Water Conservancy in Anhui Province plans to account for 82% of the total arable land in the province by the end of 2022). Due to insufficient data on the agricultural planting structure of each village, this research does not consider the planting structure, and some indicators involved are calculated based on the average value of all crops. At present, the scope of water rights trading is the water rights saved by agricultural water-saving irrigation. Taking into account the influence of factors such as precipitation, the author will also include the water yield data of each village calculated by the water yield module of the InVEST model into the tradable water volume. The direction of circulation is water trade from agriculture to industrial enterprises. Since the water demand of industrial enterprises is a long-term flow, the operating life of industrial engineering facilities is 15–25 years [56], combined with the actual situation of 52 administrative villages in Lu’an City, the time limit for water rights transactions is determined to be 25 years in this research. (According to the “Notice on Deepening the Establishment of Water-saving Irrigation Districts” (Bannong [2021] No. 107) document).

### 4.3. Water Yield

**Figure 5:**
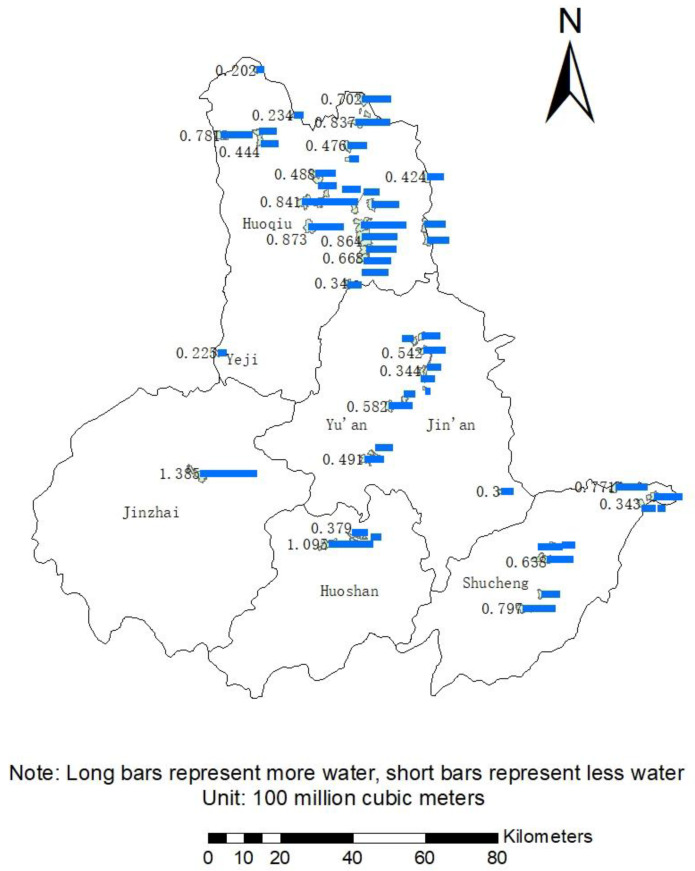
Water yield of 52 villages.

After entering the data in the InVEST model, the result after running is as shown in the Figure 5 below:

Banpeng Village in Jinzhai County produces the most water, reaching 138.5 million cubic meters, followed by Heishidu Village and Mananyuan Village in Huoshan County and Huoqiu County, respectively; The water production volume exceeds 100 million cubic meters. The selected 52 villages in Lu’an City have a total water production volume of approximately 2.731 billion cubic meters, with an average water production volume of approximately 502.5 million cubic meters per village. Among the four counties and three districts of Lu’an City, Huoqiu County produces the most water. The main reason is that Huoqiu County has the largest number of administrative villages that meet the water body exceeding 20% and the cultivated land area does not exceed 50%. More than half of these villages have above average water yield. Followed by Shucheng County and Yu’an District, the water output is 487.1 million cubic meters and 390.4 million cubic meters, respectively, accounting for 17.83% and 14.29% of the total water production in Lu’an City. Then there are Huoshan County, Jinzhai County, Jin’an District and Yeji District (in order of water production). Among them, although Jinzhai County has only one eligible village, because the village is located in the middle of Meishan Reservoir and the total area of the village is relatively large; in the land use type, the forest land covers an area of 63.64%, so it has strong water conservation. Therefore, the water yield of the village is far above the average level, which is 2.64 times the average water yield.

### 4.4. Water Saving Amount

According to the response of the water conservancy department of Anhui Province, between 2011 and 2015, the irrigation water utilization coefficient in Anhui Province increased from 0.498 to 0.515, which shows that Anhui Province has been actively carrying out farmland water-saving irrigation work, and it has been very effective. However, there is still a big gap between the national average level of 0.536, especially the world advanced level of 0.7 to 0.8. It is pointed out in the “Special Plan for the Last Kilometer of Farmland Water Conservancy in Anhui Province (2018–2022)” (hereinafter referred to as the “Special Plan”) that by the end of 2022, the province’s irrigation water utilization coefficient needs to reach 0.545. Considering the availability and completeness of the data, this research assumes that 2015 is the current level year, and the value of $\mu_{0}$ is 0.515. In addition to calculating the irrigated area based on 82% of the cultivated land area, the irrigated area of each water-saving irrigation method is determined in conjunction with the proportion of the planned and constructed irrigated area. The “Special Plan” stipulates that the low-pressure pipeline water delivery area accounts for about 6%, the sprinkler irrigation area accounts for 9%, the drip irrigation area accounts for 4%, and the remaining 81% is the soil canal water delivery and channel irrigation area. The crop irrigation water quota is obtained according to the regulations and calculation method of “Anhui Province Industry Water Quota”, and the data are shown in Table 3.

**Figure 6:**
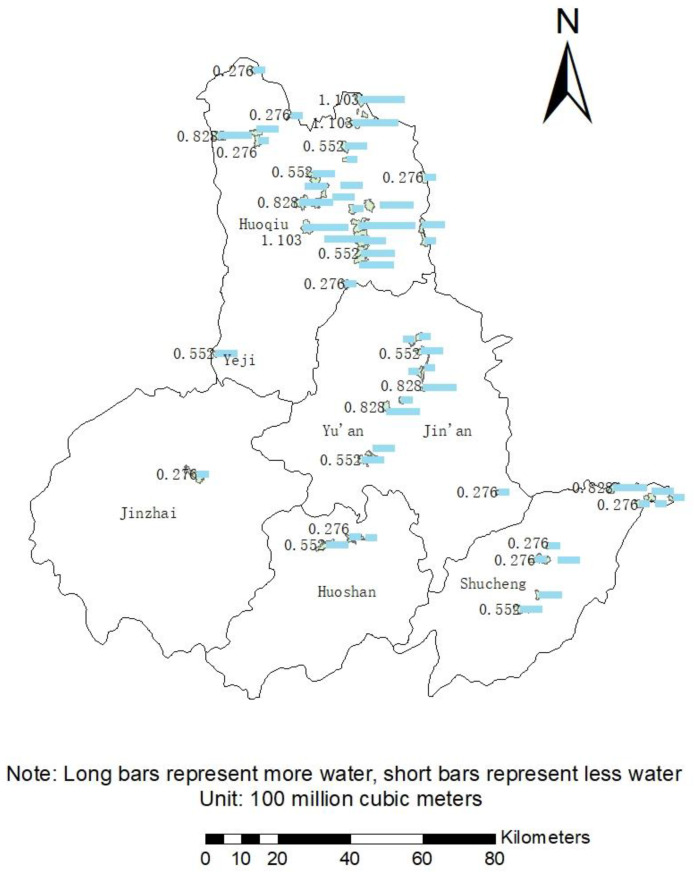
Water-saving amount in 52 villages.

Using Equation (9), it is calculated that 52 administrative villages in Lu’an City can save a total of 2.7863 billion cubic meters of water. The water-saving amount of each village is shown in Figure 6.

The average water-saving amount of 52 administrative villages is 53.6 million m^3^ per village. Nearly 30 villages have exceeded the average level of water saving. Mananyuan Village in Huoqiu County has saved the most water, saving 137.9 million cubic meters of water. Followed by Shuangzhuanjing Village, Xiajiangtai Village, Jingzhuang Village and Sanliu Village, which are located in the northeast and south of central Huoqiu County. The amount of water saved was about 110.3 million m^3^; the remaining 22 villages did not save more than the average amount of water, scattered in various districts and counties, and the amount of water saved was 227.6 million m^3^.

### 4.5. Tradable Amount of Water

**Figure 7:**
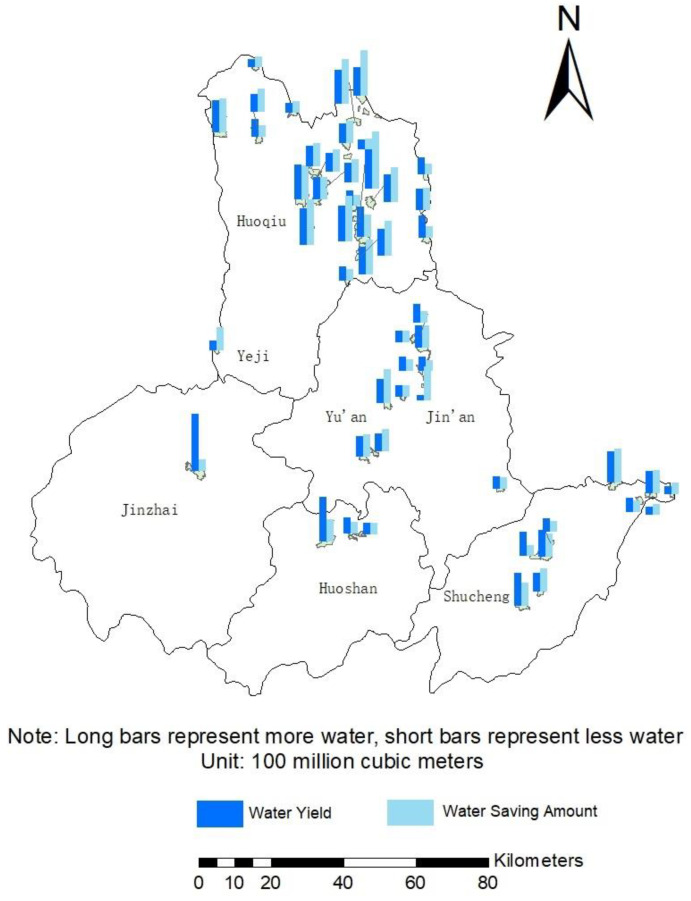
Water production and water saving amount in 52 villages.

Analyzing the water yield and water-saving amount (Figure 7), it is found that the water yield of each village is comparable to the water-saving amount. Except for the difference between the water production and water saving in Banpeng Village, Jinzhai County, which exceeds 100 million m^3^, the difference between the other villages does not exceed 70 million m^3^. Among the 52 villages, 20 villages produced more water than saved water, and the remaining 32 villages produced less water than saved water.

The calculation of Equation (10) shows that more than half of the villages (29 of them) have tradeable water greater than the average. Mananyuan Village has the most tradable water volume and is the only village with more than 140 million m^3^. There are five villages with more than 100 million m^3^ of tradable water, namely Shuangzhuanjing Village, Xiajiangtai Village, Jingzhuang Village, Mananyuan Village and Sanliu Village. Of the other 47 villages’ tradable water volume, 14 of the villages’ water production and water savings exceeded the average level; 11 villages’ water savings exceeded the average level, but the water production volume was less than the average. The water saving in three villages was less than the average, and the water production was higher than the average. The remaining 19 villages’ water production and water saving are all lower than the average.

Through the above analysis, it can be seen that there are 52 villages in Lu’an City whose arable land area does not exceed 50% and the water body area exceeds 20%. When water-saving irrigation was carried out in 52 villages, low-pressure pipelines, sprinkler irrigation and drip irrigation accounted for 6%, 9%, and 4% of the irrigated area, respectively. Taking the influence of precipitation into account, 8% of the water saved is included in the tradable water volume. The calculation result is: the total tradable water volume is about 3.0048 billion m^3^, of which the water production and water saving are 218.5 million m^3^ and 2.7863 million m^3^, respectively.

### 4.6. Willingness to Trade Water Rights after Water-Saving Irrigation

Researches on the willingness to adopt water-saving irrigation [57] and farmers’ participation in water-saving irrigation behavior [58] show that funds have the greatest impact on farmers’ adoption of water-saving irrigation technologies. In this research, the entity’s willingness to change irrigation methods was determined by comparing the costs and benefits of farmland irrigation before and after the use of water-saving irrigation. Calculating with Equations (11) and (12), it is found that even if water is not sold after water-saving irrigation, the benefits of each village can be about 30% higher than that of non-water-saving irrigation. Therefore, the entity has sufficient motivation for water-saving irrigation. The tradable water volume of each entity is different. Generally, entities with more water are willing to sell water at a lower price, but for entities with less tradable water, they are more inclined to trade at a higher price. In December 2020, the first water rights trading project in Anhui Province officially reported by the Anhui Provincial Department of Water Resources was officially listed. The transaction price in this project is 0.044 USD/m^3^, which is about 0.029 USD/m^3^ more than the current agricultural water price. This research is based on this transaction price and uses 0.0146 USD/m^3^ as the gradient of water price growth. After investigation, it was found that the price of water sold in 52 villages and the amount of water they were willing to sell showed the following relationship.

**Figure 8:**
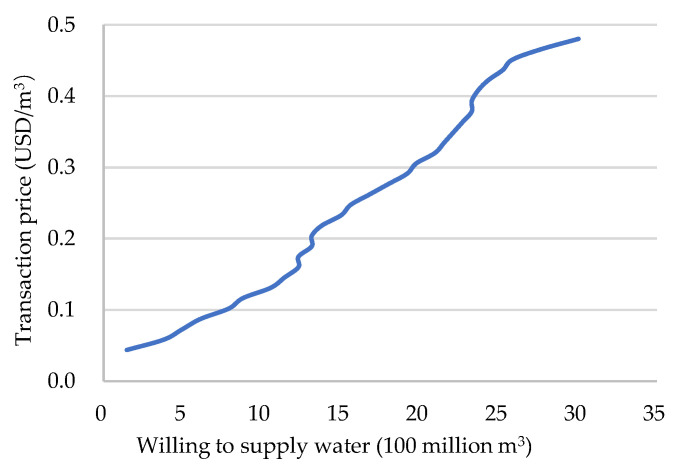
Supply curve.

It can be seen from Figure 8 that when the water price is low, village organizations are generally unwilling to participate in the transaction. As prices increase, more and more villages are willing to participate in water rights transactions. When the price increases to a certain level, all villages will participate in the transaction, and there is a positive correlation between the water price and the amount of water willing to trade. When the price remains at 0.044 USD/m^3^, only one village is willing to participate in the transaction. However, when the price rises to 0.364 USD/m^3^, all villages with more than average tradable water are willing to participate in the transaction. At this time, the total water supply is 2.2728 billion m^3^.

### 4.7. The Market-Clearing Equilibrium

Where there is demand, there will be supply. A viable water rights trading market requires that the demand curve be higher than the supply curve at the beginning. When demand increases and supply exceeds demand, prices rise. As prices rise, the supply will increase. After the quantity of supply gradually increases, market competition causes prices to fall, until the demand curve crosses the supply curve from above, which is the market clearing.

**Figure 9:**
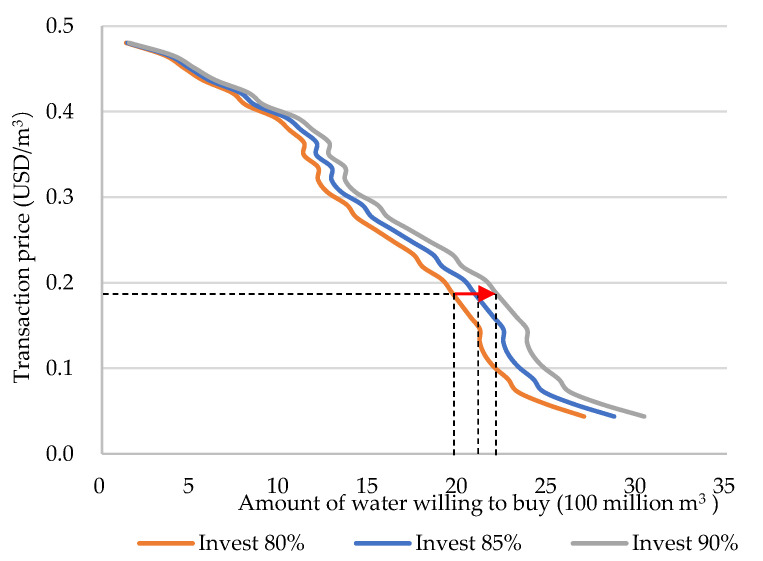
Demand curve.

The supply side can provide tradable water because most of the reason is the water saved by water-saving irrigation, in addition to the amount of water produced by the ecosystem. It is precisely because of the corresponding investment that each village has a corresponding amount of tradable water. For example, when investing USD 7.76 billion in the construction of water-saving irrigation facilities, 1.058 billion m^3^ of tradable water will be generated. When the investment in the construction of water-saving irrigation facilities increased to USD 15.31 billion, the amount of tradable water also increased to 2.097 billion m^3^. From Equation (15), it can be seen that the demand for water by a company at a certain price does not depend on the supply in the market, but is related to the cost of its own investment in water-saving irrigation. According to calculations, if the construction cost of water-saving irrigation facilities is fully borne by the enterprise, the enterprise can only purchase all the tradable water volume at a negative price, which is obviously not in line with reality, and the transaction will not occur. In the scenario where the water price is within the range of USD 0.044–0.48/m^3^ with a water price of 0.0145 USD/m^3^ as the gradient, the company’s willingness to purchase water under different proportions of the investment cost of water-saving irrigation facilities has been simulated multiple times. The results are shown in Figure 9. It shows that the higher the proportion of the company’s investment in the construction cost of water-saving irrigation facilities, the greater the demand for water at the same price.

**Figure 10:**
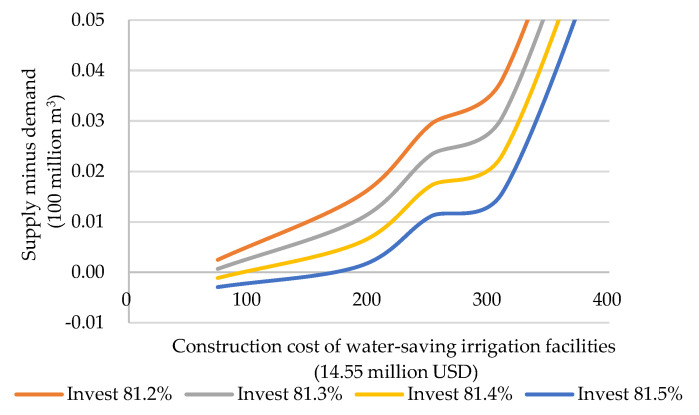
Supply minus demand under different investment ratios.

It was found in the simulation process (Figure 10) that when the company invests 81.3% of the construction cost of water-saving irrigation facilities, the supply on the market can fully meet the company’s demand under the corresponding investment. When the investment ratio is less than 81.3%, the supply on the market exceeds the demand, which is likely to cause serious investment waste of enterprises; when the investment ratio is greater than 81.3%, and if only a few villages participate in water-saving irrigation water rights transactions, the market supply cannot guarantee to meet the demand.

**Figure 11:**
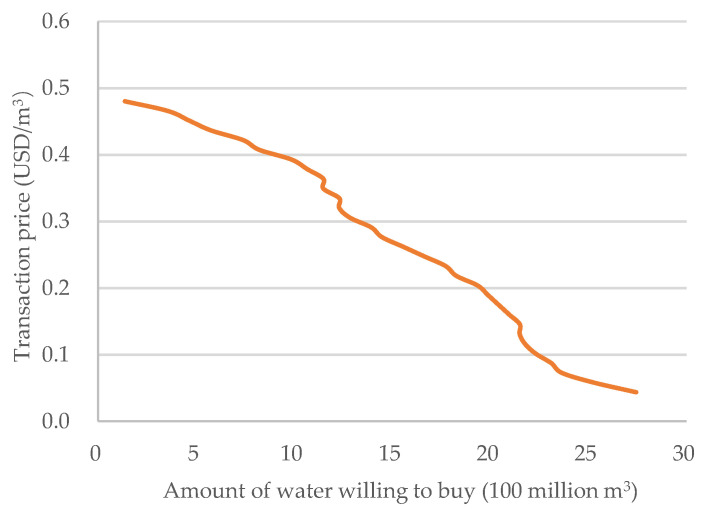
Demand curve.

The above analysis shows that, under the premise of ensuring that the company has the least investment waste and the supply can meet the demand, the company invests 81.3% of the construction cost of water-saving irrigation facilities. The demand curve is drawn as shown in Figure 11.

**Figure 12:**
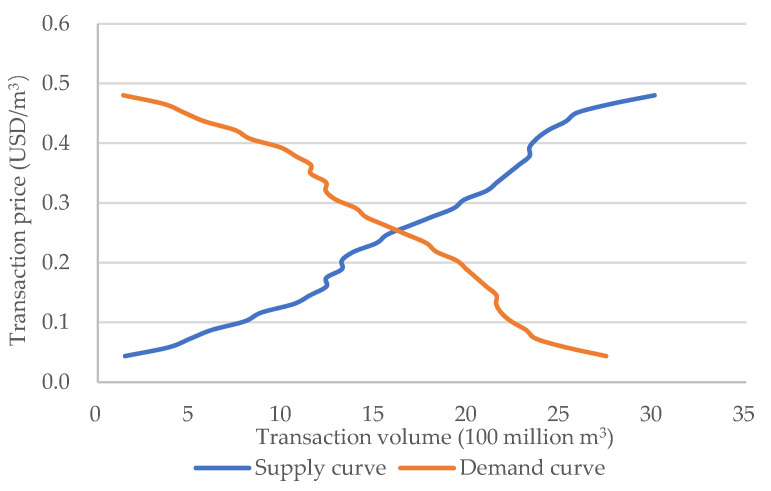
The Market-Clearing Equilibrium.

The market clearing is obtained by the intersection of the supply curve and the demand curve. As shown in Figure 12, the intersection of the demand curve and the supply curve is the equilibrium point of supply and demand, and the market reaches a short-term equilibrium state of supply and demand. Fitting the two curves separately, the equilibrium price is calculated, about 0.256 USD/m^3^, and the transaction volume is about 1.667 billion m^3^.

### 4.8. Benefit–Cost Test

When the market reached equilibrium, from the perspective of water rights sellers, 19 villages participated in water rights transactions. The water-saving irrigation project can save about 1.5725 million m^3^ of water, and 8% of the water produced is about 110.5 million m^3^. The total income of the villages that participated in the water rights transaction from selling water was USD 0.43 billion, of which five villages had more than USD 29.11 million from selling water. The cost mainly comes from the construction cost of water-saving irrigation facilities and irrigation water fees. Since the construction of public facilities like water-saving irrigation facilities in China is mainly invested by the government and enterprises, this part of the cost is almost zero for village organizations. In addition, in order to encourage efficient water-saving irrigation, the subsidy provided by the state is surplus relative to the construction cost of the project, so the main cost comes from the irrigation water fee. Due to the construction of water-saving irrigation facilities, the amount of irrigation water is reduced, so the irrigation water fee is also reduced. The calculation results show that the total saving of irrigation water costs is about USD 54.87 million, of which Mananyuan Village saves the most water costs, and the saving of irrigation water costs reached USD 4.8 million. At the same time, the village’s crop output value increased the most (about USD 0.303 billion). The crop output value of the 19 villages has increased by a total of about USD 3.46 billion, which shows that the use of water-saving irrigation can not only save water, but also increase crop production. The comprehensive cost–benefit analysis found that the total revenue of implementing efficient water-saving irrigation projects while participating in the water rights exchange is about 1.32 times that of maintaining the status quo. In addition to the advantages of saving water and increasing production, high-efficiency water-saving irrigation can also save labor, fertilizer, and seed costs. Therefore, for village organizations, the economic benefits of participating in water rights transactions and implementing water-saving irrigation are considerable.

From the perspective of the purchaser of water rights, when the market reaches equilibrium, the construction cost of water-saving irrigation facilities that companies need to invest is about USD 10.36 billion, and the total construction cost is about USD 12.72 billion. However, the actual total construction cost that needs to be invested is only about USD 12.29 billion. When the 1.667 billion m^3^ of water purchased at a price of 0.256 USD/m^3^ was used for production, the total industrial output value reached USD 28.18 billion, which is about 1.37 times the average output value at this stage. Judging from the analysis of costs and benefits, in the first year, the investment cost of enterprises in ecological infrastructure was about USD 10.77 billion, and the increase in output value was only USD 7.58 billion. However, the company’s demand for water is long-term. By the second year of the water rights transaction, the company will be able to recoup its costs. Over time, profit is inevitable. In addition, it is difficult to estimate the social impact and other benefits of enterprises investing in farmland water-saving irrigation, but they are conducive to the future development of enterprises.

From the perspective of both parties to the transaction, the village organization implements efficient water-saving irrigation with the help of the government and enterprises, and participates in water rights market transactions. Considerable economic benefits are the driving force for village organizations to implement water-saving irrigation. In addition, it is also conducive to saving water resources, saving fertilizer to protect the environment, and promoting the sustainable development of farmland. Industrial enterprises can not only obtain sufficient water resources to expand the scale of production, thereby making the enterprise profitable, but also enhance the enterprise’s sense of social responsibility and social identity. It is an intangible wealth that promotes the long-term development of industrial enterprises themselves. Industrial enterprises are willing to promote the implementation of water-saving irrigation projects. Therefore, the water-saving irrigation ecological infrastructure investment proposed in this research is theoretically feasible

## 5. Discussion

**Figure 13:**
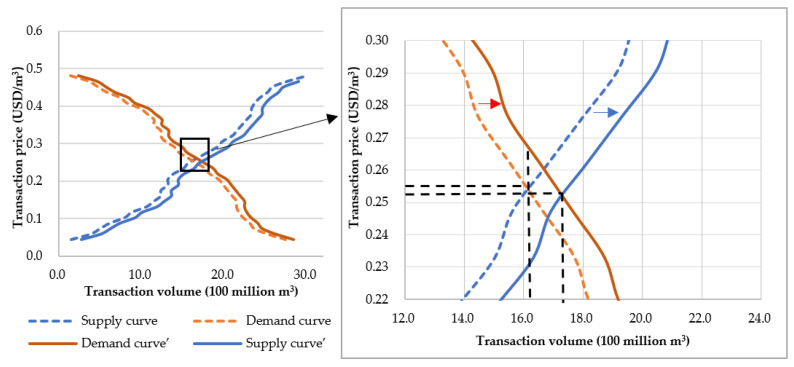
The dynamic equilibrium of market clearing (The subgraph on the right is an enlarged display of the box part of the main graph).

Combining expertise from multiple disciplines to determine market-clearing equilibrium in ex-ante benefit–cost assessments is emphasis. Generally, market-clearing is a state, which changes with changes in demand and supply. In the current equilibrium state of this case, if the demand increases by 100 million m^3^, the market is in short supply, the transaction price will rise under the same demand, and the demand curve will shift to the right (Figure 13). Then, the unit price of water will rise to 0.269 USD/m^3^. The increase in the price causes an increase in the supply. When supply exceeds demand in the market, prices will gradually fall and the supply curve will shift to the right. In dynamic changes, the market will eventually reach equilibrium again, with an equilibrium price of approximately 175.1 million m^3^ and an equilibrium quantity of approximately 1.759 billion m^3^. Similarly, when the quantity of demand decreases, the demand curve shifts to the left, supply exceeds demand, and the price drops. Therefore, the quantity of supply gradually decreases, and the price will gradually rise. Finally, supply and demand will reach a relatively equilibrium state.

Under perfectly competitive market conditions, when the market clearing equilibrium point is at the intersection of the supply curve and the demand curve, the total social efficiency is the largest, which is the Pareto optimal. At this time, an increase or decrease in output will lead to a decrease in the total efficiency of society, and a decrease in producer surplus or consumer surplus. In short, one of them will be damaged. In economics, the “rational man” assumes that the goal pursued by economic agents in decision-making is to maximize profit, that is, the marginal cost is equal to the marginal benefit. In this case, when the company intends to increase its demand by 100 million cubic meters (ΔQ=100), the exit status changes, and the final equilibrium volume increases by 92 million m^3^. In this state, the total cost (TC) of the company is USD 11.15 billion, and the total revenue (TR) is USD 28.44 billion. The marginal cost (MC) is 3.73 USD/m^3^, but the marginal benefit (MR) is 2.91 USD/m^3^. The marginal cost is greater than the marginal revenue, which means that for every additional cubic meter of water a company invests in to purchase 1 cubic meter of water for production, the increased revenue is less than the increased cost, and the company will lose money, thus reducing the purchase volume. The decrease in the demand in the market causes the price to fall, and the supply decreases accordingly. Until the marginal cost of the enterprise equals the marginal revenue, the demand reaches a relatively stable state. After that, under the influence of the market, prices and supply tend to stabilize, and the market will reach a long-term equilibrium state.

The choice of realization path is directly related to the effect of ecological product value realization. Ecological infrastructure investment has changed the number of ecosystem services, and market liquidation equilibrium reveals the value of ecosystem services. Market clearing requires the integration of expertise in multiple dimensions (such as hydrology, economy, ecology, etc.) to predict equilibrium prices and quantities, which is a huge challenge. Finding equilibrium quantities and prices begins with determining the benefits and costs of both supply and demand. Most PES projects are also ecological infrastructure construction projects, which require the demand side to be able to link cost-benefit to ecological production functions. Rather than paying for the output of ecosystem services, investing in ecological infrastructure shifts the risk of ecological production from the supply side to the demand side. This will ensure a sustainable ecosystem service output. The realization of the water rights market transaction mechanism can not only solve the problem of uneven distribution of initial water rights, but also improve the utilization efficiency of water resources and alleviate the problem of water shortages. The establishment of a water rights trading market can provide a platform for the transfer of water rights and at the same time stimulate people’s enthusiasm for water conservation. Making profits in water rights transactions is the direct driving force for the buyers and sellers of water rights to participate in market transactions. Industrial enterprises can expand their production scale from more water demand; after farmland irrigation saves water, selling the excess water can also make a profit. Due to the strong industrial water demand, it is difficult to increase the supply through the development of new water sources. Driven by industrial water demand and the potential of agricultural water-saving, it is currently the best choice to promote the transfer of agricultural water-saving water rights to industrial enterprises. Assessing water-saving irrigation infrastructure investment in irrigation districts requires a combination of knowledge from multiple disciplines. For example, the amount of water right confirmation, crop planting structure, current irrigation methods, future water-saving irrigation structure, farmers’ acceptance of water-saving irrigation, potential water saving, tradable water volume, willingness to buy and sell water rights, etc., there are differences between different transaction entities. As far as the willingness to buy and sell water rights is concerned, many scholars have carried out research on it. Through the statistics of the existing water market data, the factors leading to the occurrence of water rights transactions have been analyzed. Among them, economic benefits have the greatest impact on the willingness and behavior of water rights buying and selling [57], which is also a factor that this research focuses on and accounts for.

In addition, attention should also be paid to the external impact of market-based water rights transactions, mainly referring to the delivery of water after water rights transactions. Changing the original location and method of water use may affect the ecosystem, hydrological processes, and other water use entities that are not involved in the transaction. Countries with more mature water markets such as the United States and Australia have adopted legislation to stipulate that water rights transactions follow the principle of “no harm”. However, due to the complexity of water resources, these external influences in water rights market transactions are inevitable. From the mechanism of impact, there are direct impact, indirect impact, short-term impact, and long-term impact. Therefore, corresponding professionals are required to conduct in-depth research to ensure that water rights transactions do not endanger ecological security. Similarly, because it is a market-oriented water rights transaction, when the seller sees the benefits, it may uncontrollably save water, which will affect food security, etc. Therefore, legislation and policy constraints must be adopted to constrain the water rights trade. At present, complete marketization is difficult to practice, and government intervention is necessary.

## 6. Conclusions

This research took Lu’an City in Anhui Province as the object and evaluated the feasibility of investment in ecological infrastructure construction. First, the industry’s demand for aquatic ecological products is analyzed, and then the water-saving potential and willingness of farmland water-saving irrigation are evaluated. Finally, the equilibrium price and quantity of aquatic ecological product market transactions are obtained. The conclusions are as follows: (1) Industrial enterprises have high water efficiency, and the marginal benefit of aquatic ecological products to industry is about 6.46 USD/m^3^. (2) The construction of ecological infrastructure can increase the supply of ecological products. After carrying out water-saving irrigation of farmland, about 2.786 billion m^3^ of water can be saved. After adding 8% of the water production, the total tradable water volume is approximately 3.005 billion m^3^. (3) The profit of the entity after water-saving irrigation of farmland is at least 30% higher than the status quo. After industrial enterprises buy water, the output value can reach about 1.37 times of the current stage. Ecological infrastructure investment can generate more economic benefits. (4) The result of market clearing: 19 villages participated in water rights trading. The market equilibrium price is about 0.256 USD/m^3^, and the transaction volume is about 1.667 billion m^3^. The feasibility of investing in farmland water-saving irrigation infrastructure provides ideas for realizing the value of ecological products.

## Figures and Tables

**Table 1:** Indicators of industrial water use in Lu’an City.

Year	Industrial Output (USD 100 Million)	Fixed Assets Input (USD 100 Million)	Labor (10,000)	Industrial Water (Billion m^3^)
2010	121.97	32.47	46.8	3.41
2011	168.71	42.37	47.5	3.56
2012	221	53	48.6	3.48
2013	242.67	65.66	51.2	3.45
2014	286.45	70.51	59.65	3.18
2015	251.41	65.91	51.91	3.13
2016	249.14	58.94	54.27	2.74
2017	223.8	62.33	57.04	2.61
average	205.91	52.68	52.12	3.20

**Table 2:** Government subsidy standards for water-saving facilities.

Series	Irrigation Method	Subsidy
Water-saving facilities (1456 USD/0.667 ha)	Low pressure pipeline	0.14
	Sprinkler irrigation	0.18
	Drip irrigation	0.26

**Table 3:** Relevant data of water-saving irrigation.

Irrigation Method	Percentage of Irrigated Area %	Average Crop Irrigation Quota (m^3^/ha)	Planning Annual Irrigation Water Utilization Coefficient	Current Year Irrigation Water Utilization Coefficient
Low pressure pipeline	6	1663.71	0.545	0.515
sprinkler irrigation	9	1663.71	0.545	0.515
drip irrigation	4	1630.1	0.545	0.515

## Data Availability

The data presented in this study are available on request.
